# P283: The challenge of preventing nosocomial exposure to tuberculosis (TB) related to migrants from high incidence countries

**DOI:** 10.1186/2047-2994-2-S1-P283

**Published:** 2013-06-20

**Authors:** V Schechner, M Lidji, M Savion, G Barbash, J Abu-Hanna, T Braun, J Tarabeia, Y Carmeli, G Aviram

**Affiliations:** 1 Sourasky Medical Center, Israel; 2 Tuberculosis Prevention and Control Clinic, Tel Aviv, Israel; 3 Tel Aviv District Health Office, Tel Aviv, Israel

## Introduction

Urban hospitals in developed countries face migrants from areas highly endemic for TB. In 2011-12, it is estimated that 70,000 East African migrants settled in Tel Aviv, Israel. This was followed by a dramatic increase in the incidence of newly diagnosed TB cases in our institution (from 8 to 23 cases/10^5^ hospital days) resulting in several large scale nosocomial exposures to TB from allegedly asymptomatic patients with active TB. We intervened to protect hospitalized patients and staff from accidental exposures to TB.

## Methods

Since 28 June 2012, chest radiographs (CR) for possible pulmonary TB were performed to the high risk population visiting our center. This population was defined as persons without medical insurance who arrived from high incidence countries for TB and would stay for >24 at our hospital (admitted patients and their companions). CR were immediately categorized as low probability or possible active TB. Patients with possible TB were placed in airborne isolation and evaluated accordingly. Companions with possible TB were excluded from entry and referred for evaluation in an outpatient TB clinic.

## Results

During a 7 month period, of 1270 CR performed in high risk adult patients, 119 (9.4%) were categorized as possible TB. 83 of these were fully evaluated and pulmonary TB was confirmed in 22 patients (26%), 5 of which had no respiratory symptoms. 617 screening CR were performed in adult companions; 40 were categorized as possible TB, but only 24 were evaluated, and none of these was diagnosed with active TB. Before the intervention (Jan-June 2012) 3 events of nosocomial exposure to TB related to migrants were investigated (a total of 63 exposure days), while during the 7 months intervention period no TB exposure related to migrants required investigation.

## Conclusion

Defining high risk population among migrants and routinely evaluating them upon admission using CR is an effective strategy to prevent accidental exposures to TB in the hospital. High proportion of these patients had active pulmonary TB; many of which had no respiratory symptoms.

## Competing interest

None declared

